# Accurate Reconstruction of Image Stimuli From Human Functional Magnetic Resonance Imaging Based on the Decoding Model With Capsule Network Architecture

**DOI:** 10.3389/fninf.2018.00062

**Published:** 2018-09-20

**Authors:** Kai Qiao, Chi Zhang, Linyuan Wang, Jian Chen, Lei Zeng, Li Tong, Bin Yan

**Affiliations:** National Digital Switching System Engineering and Technological Research Center, Zhengzhou, China

**Keywords:** brain decoding, functional magnetic resonance imaging (fMRI), visual reconstruction, capsule network (CapsNet), machine learning

## Abstract

In neuroscience, all kinds of computation models were designed to answer the open question of how sensory stimuli are encoded by neurons and conversely, how sensory stimuli can be decoded from neuronal activities. Especially, functional Magnetic Resonance Imaging (fMRI) studies have made many great achievements with the rapid development of deep network computation. However, comparing with the goal of decoding orientation, position and object category from human fMRI in visual cortex, accurate reconstruction of image stimuli is a still challenging work. Current prevailing methods were composed of two independent steps, (1) decoding intermediate features from human fMRI and (2) reconstruction using the decoded intermediate features. The new concept of ‘capsule’ and ‘capsule’ based neural network were proposed recently. The ‘capsule’ represented a kind of structure containing a group of neurons to perform better feature representation. Especially, the high-level capsule’s features in the capsule network (CapsNet) contains various features of image stimuli such as semantic class, orientation, location, scale and so on, and these features can better represent the processed information inherited in the fMRI data collected in visual cortex. In this paper, a novel CapsNet architecture based visual reconstruction (CNAVR) computation model is developed to reconstruct image stimuli from human fMRI. The CNAVR is composed of linear encoding computation from capsule’s features to fMRI data and inverse reconstruction computation. In the first part, we trained the CapsNet model to obtain the non-linear mappings from images to high-level capsule’s features, and from high-level capsule’s features to images again in an end-to-end manner. In the second part, we trained the non-linear mapping from fMRI data of selected voxels to high-level capsule’s features. For a new image stimulus, we can use the method to predict the corresponding high-level capsule’s features using fMRI data, and reconstruct image stimuli with the trained reconstruction part in the CapsNet. We evaluated the proposed CNAVR method on the open dataset of handwritten digital images, and exceeded about 10% than the accuracy of all existing state-of-the-art methods on the structural similarity index (SSIM). In addition, we explained the selected voxels in specific interpretable image features to prove the effectivity and generalization of the CNAVR method.

## Introduction

Human brain decoding (Cox and Savoy, 2003; Haynes and Rees, 2006; Norman et al., 2006) plays an important role in brain-machine interfaces, may be extended to help disabled persons in expressing and motioning, and can also help us explore more about the brain mechanism. In these years, functional magnetic resonance imaging (fMRI) has become an effective tool to monitor brain activities and visual decoding based on fMRI data obtained more and more attention. In contrast to visual encoding (Kay et al., 2008) that predicts the brain activities in response to visual stimuli, the inverse decoding (Haynes and Rees, 2005; Kamitani and Tong, 2005) aims to predict the information about visual stimuli through brain activities. In general, classification, identification, and reconstruction of image stimuli based on fMRI data are three main means of visual decoding. Some progresses (Naselaris et al., 2011) have been achieved, but the most of previous researches focused on either prediction of its category (Damarla and Just, 2012; Mokhtari and Hossein-Zadeh, 2013) or identification (Kay et al., 2008) from a candidate set of image stimuli for one unknown image stimulus. The reconstruction of image stimuli is the full-information and most difficult means of decoding, and fewer studies (Naselaris et al., 2009) worked on it. The accuracy of visual reconstruction was restricted by several problems: (1) the complex noise during the acquisition of fMRI data; (2) the high dimensionality and limited number of fMRI data; (3) difficulties when imitating the human visual mechanism to develop the computation models.

Current reconstruction methods mainly focused on some simple or small image stimuli. Some methods (Thirion et al., 2006; Miyawaki et al., 2008; Van Gerven et al., 2010) directly tried to learn a linear or non-linear mapping based on limited number of samples. Thirion et al. (2006) reconstructed simple images by rotating Gabor filters in the passive viewing experiment and imagery experiment for the same subject. Miyawaki et al. (2008) achieved the reconstruction of simple binary contrast patterns (resolution: 10 × 10) by linearly mapping fMRI data to each pixel of image stimuli. Van Gerven et al. (2010) reconstructed handwritten digits ‘6’ and ‘9’ (resolution: 28 × 28) from fMRI data based on deep belief network (Hinton, 2006). Yargholi and Hossein-Zadeh (2016) employed the gauss network to reconstruct handwritten digits ‘6’ and ‘9’. Schoenmakers et al. (2013) tried to reconstruct handwritten English letters of ‘BRAINS’ (resolution: 56 × 56) from fMRI data using linear gauss model based on sparse learning. However, the direct linear mapping has limited ability to parse the complex function of visual information processing in human visual cortex, and direct non-linear mapping is easy to be overfitting based on limited number of samples and has weak generalization. So, some methods (Hardoon et al., 2004; Fujiwara et al., 2013) started to map the fMRI data to the feature representation of corresponding image stimuli, then tried to employ these features to reconstruct image stimuli. Fujiwara et al. (2013) proposed the Bayesian CCA (BCCA) model based on probabilistic extension of canonical correlation analysis (CCA) model (Hardoon et al., 2004) that related fMRI data to image stimuli via a set of latent variables. Wang et al. (2015) proposed the deep canonically correlated auto encoders (DCCAE) with a non-linear observation model, and reconstruct each view using learned representations.

With powerful feature representation, the convolutional neural network (CNN) architecture (Simonyan and Zisserman, 2014; He et al., 2016) has driven rapid development in visual computing area. Some work (Yamins et al., 2013, 2014; Kriegeskorte, 2015) has proved that features of some layers in CNN behaved strong correlation with the brain activities of particular visual cortex, and the current state of the art visual reconstruction methods relied on the mapping from fMRI data to the features of specific layer in CNN architecture, then tried to reconstruct image stimuli based on information stream of CNN architecture. Using the convolution features of the first layer in CNN, Wen et al. (2017) implemented the reconstruction of dynamic video frame by frame, and proposed two-stage cascade neural decoding method based on multivariate linear regression and deconvolutional neural network (Zeiler et al., 2011). They first predicted features by multivariate linear regression, then reconstructed images by feeding the estimated features in the pretrained deconvolutional neural network. Du et al. (2017) presented a deep generative multi-view model (DGMM) to regard the visual reconstruction as the Bayesian inference of the missing view. These studies have suggested that DNN especially CNN could help interpret human brain visual information. However, the accurate reconstruction of image stimuli remains to be challenging, and is CNN architecture the path for solving the reconstruction of image stimuli?

**FIGURE 1:**
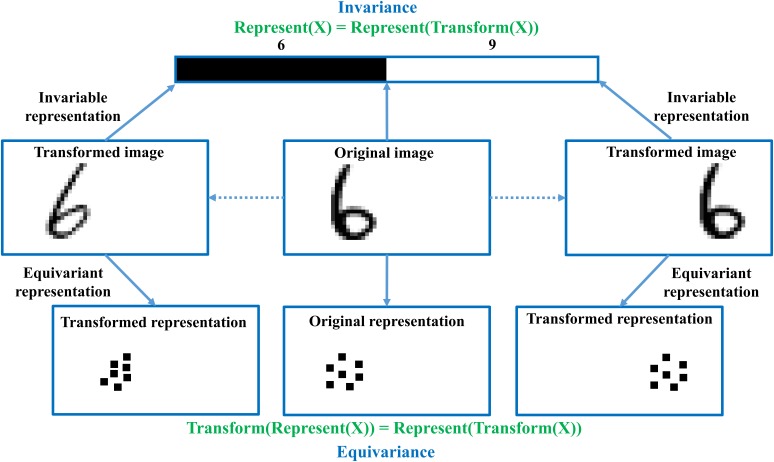
The difference between invariance and equivariance. Invariance performs the invariable representation when transforming location, pose, and orientation of digit ‘6’, in order to keep invariable class. Equivariance performs the transformed representation when transforming the location, pose, and orientation of digit ‘6’, in order to keep equivariant representation. Invariance is usually designed for the specific task, but equivariance can be used for many tasks, because the equivariant representation contains more various features about the image.

We analyzed the problem from the perspective of invariance and equivariance. Invariance and equivariance are two very important perceptions in visual representation. As shown in the **Figure 1**, the invariance is usually designed for the specific task such as the semantic extracting, at the cost of discarding other features that are not correlated with the semantic information. However, the equivariance that keeps the quantity of information unchanged while feature representation can be designed for many tasks, because it keeps various feature information such as location, pose, orientation, and so on. Essentially, CNN architecture composed of hierarchical convolutional and pooling layers, was firstly designed for invariance including translation invariance, rotating invariance, scale invariance, and so on. During the forward propagation in CNN architecture, extracted features are more and more abstract and local detailed information such as pose and location which are valuable for detection or segmentation is sacrificed, which lead to the difference of performance (Jia et al., 2009) on classification (Krizhevsky et al., 2012) and detection (Girshick et al., 2014) or segmentation (Long et al., 2015) in computer vision area. In neuroscience area, CNN can be usually designed for specific procedure of information processing in human visual cortex, such as semantic extracting, or decoding of space location, however, CNN cannot extract various features of image stimuli, thus incapable of accurate reconstruction that requires various sensory information extracting such as semantic class, orientation, location, scale, and so on.

In addition, we analyzed it from the perspective of human visual mechanism. As we know, after one person glances at one image, he or she can simultaneously answer many questions about the image such as ‘what is the main object?’, ‘where is the object?’, ‘what characteristics does the object have?,’ and so on. We noticed that these questions contain many characteristics of the image and we can conclude that the procedure of visual information representation in human visual cortex requires the equivariance instead of invariance, because only equivariance can ensure that the semantic class, location, scale, orientation and some other detailed information be preserved instead of discarding, in order to efficiently deal with various visual tasks.

According to the above analysis, in order to achieve accurate visual reconstruction, we need an architecture that can keep the equivariance when feature representation and contain various features to make it possible to achieve accurate reconstruction from the perspective of information completeness.

Aimed at the equivariance instead of simple invariance, Sabour et al. (2017) firstly proposed the concept of capsule and designed the promising capsule network (CapsNet) based on convolutional operation and routing by agreement. In CNN architecture, each layer just includes some disorder neurons which makes it hard to perform some organizations of detailed internal structure. However, in the CapsNet, capsules serve as the basic units of each layer and contain a group of neurons, which can organize some internal structures inspired by the structure of cortical mini column (Buxhoeveden and Casanova, 2002) including several hundred neurons in primates. The length of a capsule’s features can predict the presence of a particular object for the invariance, and the capsule’s features can predict the various attributes of a particular object for the equivariance. The CapsNet reached high accuracy on MNIST (LeCun, 1998) digits recognition and reconstruction, which benefits from the equivariance when feature representation. The CapsNet can achieve accurate classification, which can prove that the capsule’s features contained abstract information; can achieve accurate reconstruction, which can prove that the capsule’s features contained various information of image stimuli and is the equivariance of image stimuli.

In this study, our main contributions are as follows: (1) we introduced the concept of invariance and equivariance to analyze the disadvantage of previous CNN on visual reconstruction compared to the new CapsNet architecture; (2) we proposed the new CapsNet architecture based visual reconstruction (CNAVR) method that accords well with the human visual information representation in human visual cortex based on the equivariance; (3) we interpreted the selected voxels used to reconstruct image stimuli in specific interpretable features; (4) this paper is the first to study visual reconstruction via the new promising CapsNet architecture.

## Materials and Methods

### Experiment Data

We employed the dataset from Van Gerven et al. (2010) in the study. A hundred handwritten gray-scale digits (equal number of ‘6’ and ‘9’) at a 28 × 28 pixel resolution taken from the MNIST database were presented to one subject. In each trial, a handwritten digit ‘6’ or ‘9’ was presented to the subject, remained visible for 12.5 s, and flickered at a rate of 6 Hz on a black background. There are four runs interspersed with 30 s rest periods to perform 100 trials in total, and trials in the same run were separated by a 12.5 s interval. The Siemens 3T MRI system was used to acquire blood-oxygenation-level dependent (BOLD) sensitive functional images. The single-shot gradient EPI sequence with a repetition time (TR) of 2.5 s and isotropic voxel size of 2 × 2 × 2 mm^3^ was employed. The functional images were acquired from 10 to 15 s after trial onset and averaged to obtain an estimate of the steady-state response. The acquired data was pre-processed including motion-corrected, coregistered with the anatomical scan, detrended, and high-pass-filtered dealing with hemodynamic response function (HRF) issue within Statistical Parameter Mapping (SPM5) software. The fMRI data of each image stimulus contains 3,092 voxels in total from V1, V2, and V3 regions. Additional, the detailed information about the fMRI data can refer to Van Gerven et al. (2010), and the public dataset can be downloaded through^1^. In the experiment, we employed 10-fold cross validation to test the CNAVR method.

### The Overview of CNAVR Method

**FIGURE 2:**
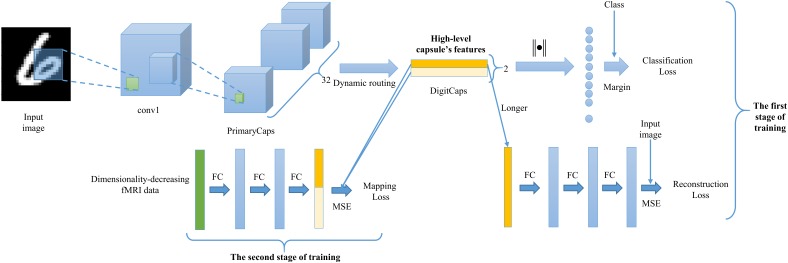
The proposed CNAVR method. The first stage of training aims to construct the equivariance of feature representation between image stimuli and high-level capsule’s features. The second stage of training aims to construct the mapping from the fMRI data to the corresponding capsule’s features for digits ‘6’ and ‘9’. The reconstruction can be achieved by using the longer capsule’s features that represent the various features about the image stimuli.

In order to achieve accurate visual reconstruction, as analyzed in the introduction about the equivariance and invariance, we employed the new CapsNet architecture (Sabour et al., 2017) to construct the feature representation of the equivariance between image stimuli and capsule’s features, and learned the mapping from fMRI data to capsule’s features based on several fully connected neural network. As shown in the **Figure 2**. Our proposed CNAVR method included two-stage training. Firstly, we employed the CapsNet to train the equivariance from images to capsule’s features, and from capsule’s features to images in an end-to-end manner by using convolutional, fully connected, and routing by agreement operations. After the training, given one input image, we can obtain the corresponding high-level capsule’s features, and can reconstruct the input image accurately again based on the capsule’s features, which indicated that the capsule’s features did not throw away location, pose, scale characteristics and so on for the sake of invariance, and kept complete information of the image when feature representation. Then we selected voxels to reduce dimensionality of fMRI data by encoding performance with capsule’s features, and learned the mapping from dimensionality-decreasing fMRI data to the high-level capsule’s features using three layers’ fully connected neural network. After the two-stage training, given the fMRI data of one presented image stimulus, we can predict its high-level capsule’s features about digits ‘6’ and ‘9’ with the learned mapping, and the accurate reconstruction can be accomplished using the longer capsule.

It should be noted that next section “Capsule and Dynamic Routing Between Capsules” and “Training Image Feature Representation of Equivariance” simply introduced the basic concept of capsule unit and the CapsNet architecture respectively to make it easy to understand our CNAVR method, and more detailed information about the CapsNet can refer to Sabour et al. (2017). The section “Selecting Voxels by Encoding Performance to Decrease Dimensionality of fMRI Data” demonstrated how to select valuable voxels to reduce the dimensionality of fMRI data. How to train the mapping from the dimensionality-decreasing fMRI data to the high-level capsule’s features was illustrated in the section “Training the Mapping From Dimensionality-Decreasing fMRI Data to High-Level Capsule’s Features,” and the section “Reconstructing Image Stimuli From Human fMRI” explained how to use previous trained network in the section “Training Image Feature Representation of Equivariance,” and “Training the Mapping From Dimensionality-Decreasing fMRI Data to High-Level Capsule’s Features” to accomplish reconstruction from human fMRI.

### Capsule and Dynamic Routing Between Capsules

Sabour et al. (2017) recently proposed the concept of capsule and dynamic routing between capsules. Each capsule contains a group of neurons. As the equation (1), the capsule *j* performs the non-linear squashing activation function for the given input vector **s***_j_*, and output vector **v***_j_*. The orientation of vector **s***_j_* is preserved, but the length is squashed between 0 and 1. The parameters in **v***_j_* represent the various properties of a particular entity such as position, scale, and texture, and the length of **v***_j_* is used to represent the existence of the entity.

(1) $$v_{j}=\frac{{\left\|s_{j}\right\|}^{2}}{1+{\left\|s_{j}\right\|}^{2}}\frac{s_{j}}{{\left\|s_{j}\right\|}^{2}}$$

The input **s***_j_* is a weighted sum over all prediction vectors **û***_j_*_∣_*_i_* that is produced by multiplying the output **u***_i_* of a capsule in the layer below by a weight matrix **W***_ij_*.

(2) $${\hat{u}}_{j|i}=W_{\mathrm{ij}}u_{i}$$

(3) $$s_{j}=\sum_{i}c_{\mathrm{ij}}{\hat{u}}_{j|i}$$

The coupling coefficients *c_ij_* are determined by the iterative dynamic routing process. The coupling coefficients between capsule *i* and all the capsules in the layer above are determined by the softmax of *b_ij_* indicating the probability that capsule *i* should be coupled to capsule *j*.

(4) $$b_{\mathrm{ij}}=b_{\mathrm{ij}}+{\hat{u}}_{j|i}v_{j}$$

(5) $$c_{\mathrm{ij}}=\frac{\exp(b_{\mathrm{ij}})}{\sum_{k}\exp(b_{\mathrm{ij}})}$$

Where *b_ij_* is initially set to zero, then is iteratively refined by measuring the agreement between the output **v***_j_* and the prediction û*_j_*_∣_*_i_* made by capsule *i* in the layer below, using the scalar product **v***_j_***û***_j_*_∣_*_i_*. Three looping can obtain the nice coupling coefficients and routing by agreement essentially tried to learn the relationship between part and whole.

### Training Image Feature Representation of Equivariance

**FIGURE 3:**
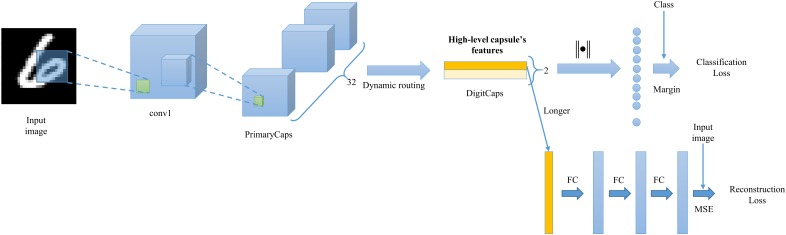
The architecture of CapsNet. The first two layers perform convolutional operations to construct the primary capsule structure. Each capsule in the PrimaryCaps layer includes 8D features, and each high-level capsule in the DigitCaps layer includes 16D features that include more various features. The architecture employs the dynamic routing to replace the pooling operations to avoid the loss of the valuable information such as orientation, location, scale, and other detailed features that are important for the equivariance when forward propagation. The ‘deep yellow’ mark represents the capsule for digit ‘6’ in the DigitCaps and using it can predict the input image based on three fully connected layers.

We employed the CapsNet (Sabour et al., 2017) to train the equivariance between images and corresponding high-level capsule’s features using handwritten digit images from LeCun (1998). As shown in **Figure 3**, the CapsNet can be divided into three parts. The first part (feature representation part) is used to extract image features, the second part (classification part) is used to classify the input image with the extracted features, and the third part (reconstruction part) is used to reconstruct images again with the extracted features. In detail, the CapsNet architecture is shallow with only two convolutional layers, dynamic routing layers and several fully connected layers. Given one image (size: 28 × 28), the first layer (Conv1) performs 256 convolutional (kernels: 9 × 9) operations with a stride of 1 and ReLU (Szegedy et al., 2015) activation. This layer converts pixel intensities to the local features (size: 20 × 20) that are then used as inputs to the primary capsules. The second layer (PrimaryCaps) also performs 256 (32 × 8) convolutional (kernels: 256 × 9 × 9) operations with a stride of 2 to produce 32 capsule maps (size: 6 × 6) whose capsule is an 8D vector. This layer is to construct capsules for dynamic routing operation in next layer. The final Layer (DigitCaps) has one 16D capsule per digit class (6 and 9) and each of these capsules receives input from all 1152 (32 × 6 × 6) capsules in the layer below.

Because the length of each capsule represents the probability that a specific entity exists, the CapsNet aims to make the corresponding high-level capsule vector longer if some digit is present in the image and make the other high-level capsule vectors shorter. So, the classification loss is simply the sum of the losses of two digit capsules, and defined as shown below. In the training, set T_C_ = 1 if a digit of class c is present and set m^+^ = 0.9, and m^−^ = 0.1 and *λ* = 0.5.

(6) $$L_{\mathrm{Classification}}=\sum_{c}\left(T_{c}\max(0,m^{+}-{\left\|v_{c}\right\|}^{2}+\lambda (1-T_{c})\max{\left(0,\left\|v_{c}\right\|-m^{-}\right)}^{2}\right)$$

Most importantly, in order to ensure the equivariance of mapping from images to high-level capsule’s features, the CapsNet adds a decoding network (reconstruction part) on the top of the capsule network. The decoding network contains three fully connected layers using ReLU activation function in the first two layer and sigmoid activation function in the output layer. The reconstruction can be accomplished with the corresponding capsule’s features. The full valuable various information for image reconstruction is preserved in the high-level capsule’s features by minimizing the mean squared error (MSE) between the reconstructed image and the input image.

So, the overall loss _L_Overall__ is the classification loss plus the weighted decoding loss. The classification loss is to force the high-level capsules distinct in the length for different digits. The reconstruction loss is to force the network to preserve all the information required to reconstruct the image throughout the CapsNet, and acts a bit like a regularization.

(7) $$L_{\mathrm{Overall}}=L_{\mathrm{Classification}}+\mu \mathrm{MSE}(I,{\mathrm{FC}}_{\mathrm{decoding}}(v_{k}))$$

In the experiment, the Adam optimizing (Kingma and Ba, 2014) was used to avoid overfitting and fasten the training. It is noted that was set 4.0 and batch size was set 10. We finished the training after about 20 epoch based on the MNIST dataset using Tensorflow (Abadi et al., 2016).

### Selecting Voxels by Encoding Performance to Decrease Dimensionality of fMRI Data

After finishing the training of the CapsNet, we obtained the architecture of feature representation for equivariance instead of invariance. Given one image stimulus, we can predict its two high-level capsule’s features and also can reconstruct the image using the longer 16D capsule’s features. In order to realize the reconstruction of image stimuli from fMRI data, we need to map fMRI data to high-level capsule’s features. However, the amount of fMRI data is usually limited because of the acquiring device, subjects, time and other reasons, and the dataset used in this study only contains 100-pair digits (50 digits ‘6’ and 50 digits ‘9’) and corresponding fMRI data in total. Moreover, the dimensionality of the fMRI data reaches 3,092, a very high number compared to the number of the samples, which easily lead to overfitting during training.

**FIGURE 4:**
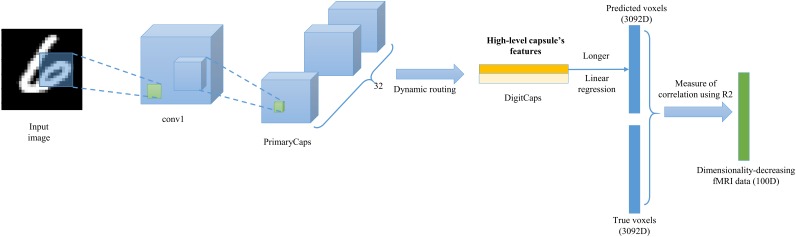
The encoding architecture used to select valuable voxels. The simple linear regression is used to fit to the each of voxels (3092D) using the longer of two high-level capsule’s features. The encoding performances are measured through R2. According to the R2 values of each linear regression for each voxel, those voxels whose R2 values belong to top-100 are selected and used to perform next reconstruction.

Faced with the problem of high dimensionality, we chose to use those voxels that are maximally correlated with the image stimuli and the correlation is measured by the effect of fitting namely encoding performance. So, we built encoding model mapping image stimuli to voxels. As shown in the **Figure 4**, given one image stimuli, we can predict its two (digits ‘6’ and ‘9’) high-level capsule’s features based on the trained CapsNet in the section “Training Image Feature Representation of Equivariance”, and selected the longer capsule, then employed simple linear regression to learn to fit each voxel using the capsule’s features that are the equivariance of image representation, contain all valuable information and have the shorter dimensionality. During fitting each voxel using linear regression, we can measure the encoding performance of each fMRI voxel using the coefficient of determination (R2), which indicates the percentage of variance that is explained by the model. Finally we selected those voxels whose R2 is at the top 100, and reduced the dimensionality of fMRI data from 3092 to 100.

### Training the Mapping From Dimensionality-Decreasing fMRI Data to High-Level Capsule’s Features

**FIGURE 5:**
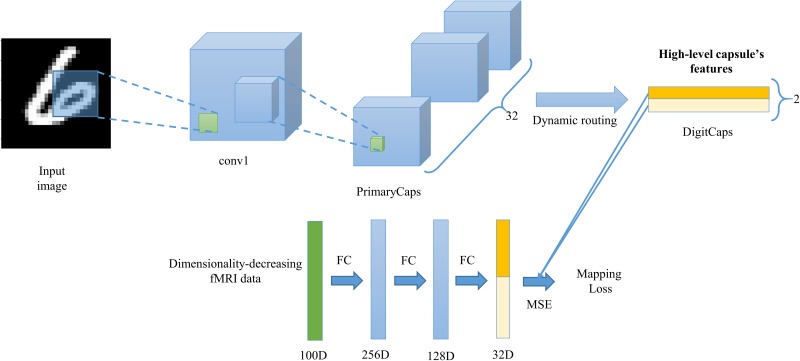
The architecture that maps the fMRI data to capsule’s features. The three fully connected layers are employed to predict ‘pale yellow’ capsule and ‘deep yellow’ capsule that represent digit class ‘9’ and ‘6’ respectively. The ‘cyan’ vector represents the dimensionality-decreasing fMRI data based on the encoding performance that evaluated by the linear regression of high-level capsule’s features. The trained CapsNet in **Figure 3** is used to make up training samples (pairs of two capsule’s features and dimensionality-decreasing fMRI data) to train the mapping from the fMRI data to capsule’s features.

Next, as shown in the **Figure 5**, we designed the network that maps the dimensionality-decreasing fMRI data to the two capsules of digits ‘6’ and ‘9’. Our network was composed of three fully connected layer using ReLU activation function in the first two layer and no activation function in the last layer. In the experiment, we tried to add the number of layers, but find no benefit. The output of first layer was 256D, the second 128D, and the last 32D. We added the L2 regularization operations in the first two layers to prevent the network from overfitting because the number of training samples is too limited to be easily overfitting. The output of last layer was split into two 16D vectors in the middle and employed squashing function to resize each length between 0 and 1. The two 16D vectors represent the prediction of high-level capsule’s features. The true high-level capsule’s features can be obtained based on the feature representation part of trained CapsNet in the “Training Image Feature Representation of Equivariance”. We employed the mean square error (MSE) between predicted and true capsule’s features to perform gradient descent to update the weight parameters of the three layer’s neural network. It should be noted that the weights in the CapsNet is fixed when training the three layers’ network.

**FIGURE 6:**
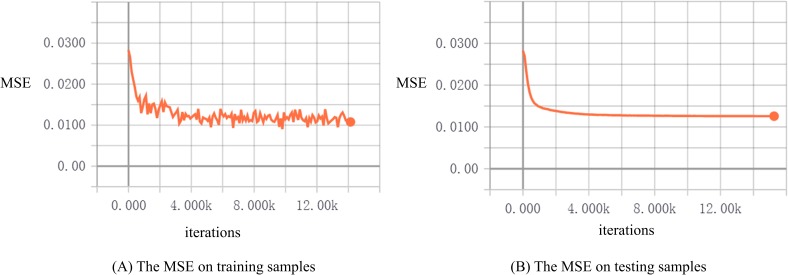
The learning curves during training. The mapping from fMRI data to high-level capsule’s features is important for next reconstruction. The MSE on testing samples is close to that on the training samples, which indicates that our proposed CNAVR method avoided the overfitting, because of the dimensionality-decreasing operations and the capsules including the feature information of equivariance. The performance will get improved when obtaining more fMRI data.

We employed the Adam optimizing method to perform the training. The batch size is set 10, initial learning rate is set 0.0001. The learning curve of the training was shown in the **Figure 6**. We finished the training after about 10,000 iterations using Tensorflow, and we can see that our network behaves well on the 90 training samples and do not suffer from the overfitting because of the regularization and voxels selecting operations. So far, we accomplished the mapping from the dimensionality-decreasing fMRI data to high-level capsule’s features.

### Reconstructing Image Stimuli From Human fMRI

**FIGURE 7:**
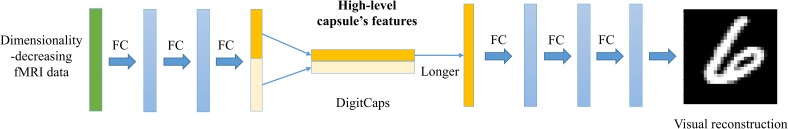
The flow of visual reconstruction. The former half part trained at the second training stage is responsible for the mapping from dimensionality-decreasing fMRI data to the two high-level capsule’s features, the latter part (reconstruction part) trained at the first stage is responsible for the reconstruction based on the longer capsule. In this way, the high-level capsule’s features are used to bridge between fMRI data and image stimuli.

After the two-stage training above, we accomplished the equivariance between images and capsule’s features in the “Training Image Feature Representation of Equivariance”, and the mapping from the dimensionality-decreasing fMRI data to high-level capsule’s features in the section “Training the Mapping From Dimensionality-Decreasing fMRI Data to High-Level Capsule’s Features”. So, we can obtain our CNAVR model and reconstruct the image stimuli using dimensionality-decreasing fMRI data. As show in the **Figure 7**, given one fMRI vector, we firstly selected the valuable voxels according to the encoding performance in the section “Selecting Voxels by Encoding Performance to Decrease Dimensionality of fMRI Data,” and predicted its two high-level capsule’s features about the digits ‘6’ and ‘9’ based on the trained three layer’ neural network in the section “Training the Mapping From Dimensionality-Decreasing fMRI Data to High-Level Capsule’s Features,” secondly we take out the longer capsule, finally we can accomplish accurate reconstruction by the mapping from high-level capsule’s features to images based on the reconstruction part of trained CapsNet in the section “Training Image Feature Representation of Equivariance”.

## Results

### The Encoding Performance

**FIGURE 8:**
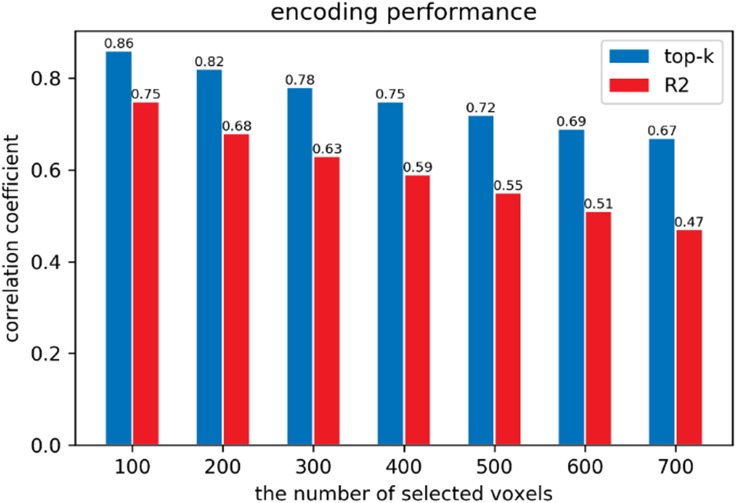
The encoding performance according to different methods of selecting. The X-axis represents the number of voxels selected from 3,092 voxels. The Y-axis represents the mean correlation coefficient of prediction on testing set for selected voxels by 10-fold cross validation. We can see that employing the high-level capsule’s features can achieve good encoding performance, which indicates that these capsules include various features such as semantic class, orientation, location, and so on.

The dimensionality (3092D) of the fMRI data is too big to train the mapping from fMRI data to the high-level capsule’s features using limited number (90) of samples, so we selected some valuable voxels according to coefficient of determination R2 reflecting the performance of fitting on training set. As shown in the **Figure 8**, we employed the 10-fold cross validation to test our encoding performance for each voxel. We can see that mean correlation coefficient of top-100 voxels reached 0.86 and that of top-700 voxels exceeded more than 0.65. The performance indicated that the capsule’s features make for better encoding although using the simplest linear regression, which proved the advantage of equivariance of CapsNet when feature representation. In addition, we found that we nearly selected the most of those top voxels using R2, and the selected voxels reached nearly about 85% of top-k voxels in terms of mean correlation coefficient. The comparison demonstrated that selecting voxels by R2 is a good choice and ensures the performance of the next visual reconstruction.

### The Results of Reconstruction

We employed several standard image similarity metrics, including Pearson correlation coefficient (PCC), mean squared error (MSE), and structural similarity index (SSIM) (Wang et al., 2004). Note that MSE and PCC is not highly indicative of similarity, and serves as the auxiliary metrics, while SSIM proposed to measure structural similarity, can address this shortcoming by taking texture into account and has strong persuasion.

Firstly, we presented the reconstruction results of 12 distinct handwritten digits including the equal number of digits ‘6’ and ‘9’. In order to present the results clearly, we gave image stimuli, the theoretical reconstruction based on the true high-level capsule’s features of image stimuli through trained CapsNet in section “Training Image Feature Representation of Equivariance”, and the visual reconstruction based on the predicted high-level capsule’s features of fMRI data through trained three layers’ neural network in section “Training the Mapping From Dimensionality-Decreasing fMRI Data to High-Level Capsule’s Features”. The theoretical reconstruction demonstrates the theoretical upper limit of our CNAVR method.

**FIGURE 9:**
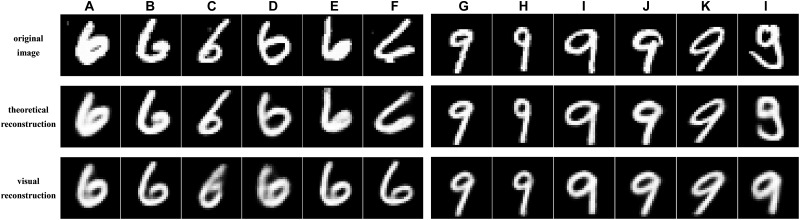
Reconstruction results of the proposed CNAVR method. The presented reconstruction both includes five high-quality and one low-quality results for digits ‘6’ and ‘9’. Except for the ‘f’ and ‘l’ columns, the reconstruction gets close to original image stimuli, from the view of orientation, scale, location, and so on. The ‘f’ and ‘l’ columns represent the small number of low-quality reconstruction, and we can see that the most of low-quality reconstruction belong to the strange image stimuli, which needs further specific improvement.

From the **Figure 9**, we can see that the theoretical reconstruction is perfect and much close to the image stimuli, because capsule’s features guarantees no missing information when feature representation through trained CapsNet, which proved the equivariance. In addition, our visual reconstruction results are also much similar with image stimuli, which proved the proposed CNAVR method. In detailed, we gave corresponding quantitative evaluation for each reconstruction in **Table 1**. It cannot be denied that some image reconstruction is not good, as shown in the column ‘f’ and ‘l’ in the **Figure 9**. We analyzed that the second stage of mapping from voxels to capsule’s features remained improvement based on limited fMRI data, and the reconstruction part in the CapsNet is sensitive to the input capsule, and reconstruction results will change when the high-level capsule’s features are slightly perturbed. In addition, we can see that these image stimuli that cannot be reconstructed accurately indeed do not belong to the common digits. The subject may recall corresponding common pattern if a common subject suddenly looks at the strange kind of image stimuli, which may be an interesting question.

**Table 1:** The corresponding quantitative evaluation for each presented reconstruction in **Figure 9**.

Metrics	a	b	c	d	e	f	g	h	i	j	k	l
MSE	0.023	0.021	0.029	0.037	0.048	0.090	0.014	0.013	0.022	0.026	0.024	0.115
PCC	0.934	0.917	0.833	0.832	0.774	0.521	0.912	0.900	0.890	0.873	0.869	0.460
SSIM	0.906	0.885	0.826	0.826	0.772	0.516	0.901	0.898	0.888	0.867	0.866	0.459

Next, we presented the quantitative results based on 10-fold cross validate compared to the several state of the art methods. As shown in the **Table 2** below, although the PCC and MSE of our proposed CNAVR method is a little weaker than the current best DGMM (Yamins et al., 2014) and De-CNN (Wen et al., 2017), the CNAVR exceeds about 10% than them on more important SSIM metric. We analyzed that the methods should not be crazy about the much high MSE and PCC, because the complex noise in the fMRI data and limited samples reduce the significance of pixel-level comparison. Moreover, human do not care much detailed information in pixel-level and should care much of structure according to attention mechanism. So, the two metrics just serve as the auxiliary measure, and our CNAVR performed better overall.

**Table 2:** The quantitative comparison to other state of the art methods.

Algorithms	MSE	PCC	SSIM
Miyawaki et al. (2008)	0.042	0.767	0.466
Fujiwara et al. (2013)	0.119	0.411	0.192
Wang et al. (2015)	0.074	0.548	0.358
Wen et al. (2017)	0.038	0.799	0.613
Du et al. (2017)	**0.037**	**0.803**	0.645
Our CNAVR	0.042	0.769	**0.750**

*The bolded values represented the best performance on the corresponding metrics.*

### Demonstration of Voxels Related to Specific Features

Further, we presented the relationship between voxels and specific interpretable features to make the proposed CNAVR method interpretable and prove its generalization. In order to interpret the selected voxels and make clear whether some voxels can explicitly influence the appearance of reconstructed images, such as shape, orientation and so on, we proposed to explain voxels in specific features based on the gradient information of the CNAVR network.

**FIGURE 10:**
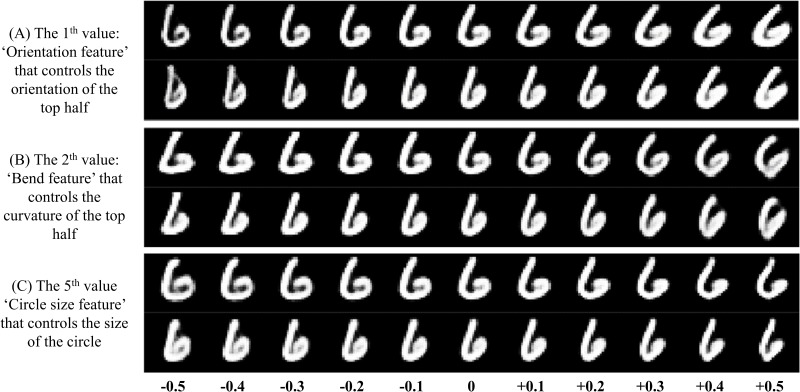
Some specific interpretable features for digit ‘6’. The three interpretable features including **(A)** ‘Orientation feature’, **(B)** ‘Bend feature’, and **(C)** ‘Circle size feature’ are presented. For example, in panel **(A)**, the first value in the 16D feature vector can control the orientation of the top half for digit ‘6’, and is called as ‘Orientation feature’. In this way, we can interpret the abstract numerical value with the image features.

**FIGURE 11:**
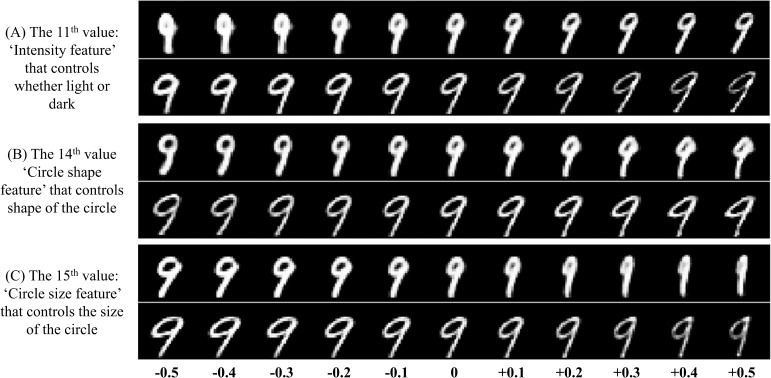
Some specific interpretable features for digit ‘9’. The three interpretable features including **(A)** ‘Intensity feature’, **(B)** ‘Circle shape feature’, and **(C)** ‘Circle size feature’ are presented. For example, in panel **(A)**, the eleventh value in the 16D feature vector can control whether light or dark for digit ‘9’, and is called as ‘Intensity feature’. In this way, we can interpret the abstract numerical value with the image features.

**FIGURE 12:**
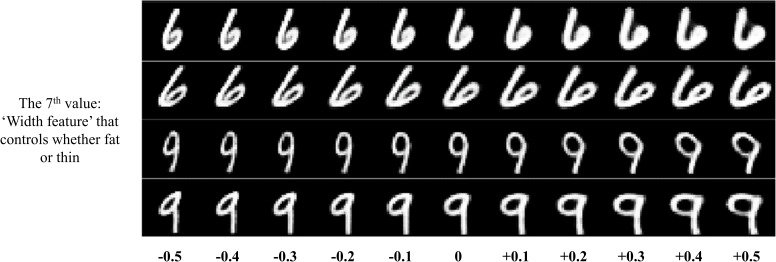
The sharable interpretable features for both digits ‘6’ and ‘9’. The seventh value can influence whether fat or thin for both the digits ‘6’ and ‘9’, which shows that the information between digit ‘6’ and ‘9’ can be generalized in the reconstruction part in the CapsNet.

**FIGURE 13:**
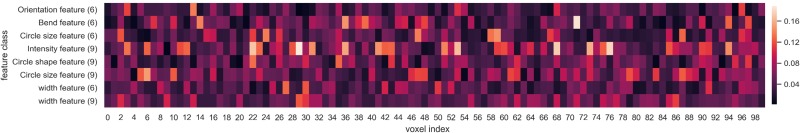
Heat map illustrating the relationship between voxels and specific interpretable features. The contribution of the specific voxels to the specific interpretable features is presented, which makes the voxel interpretable. Different colors are used to distinguish whether specific voxels are more active than others for specific image based features.

Firstly, we slightly modified one specific value in the 16D high level capsule’s feature vector at a time and observed the corresponding transformation of reconstructed images. In this way, we found that some specific dimensions in the 16D vector can be interpretable. As shown in the **Figure 10**, the presented specific values in the capsule’s feature vector can indeed control the appearance of reconstruction for digits ‘6’. For example in the **Figure 10** (A), when we modified the 1th value in the 16D feature vector from -0.5 to +0.5, we can see that the orientation of the top half of digit ‘6’ also changes continuously. So, we call the 1th value in the 16D vector as the ‘Orientation feature’. In the same way, we presented some interpretable features for digits ‘6’. The same phenomenon about digit ‘9’ can be also seen in the **Figure 11**. In comparison between the **Figures 10**, **11**, the same interpretable features (for example ‘Bend feature’) of ‘6’ and ‘9’ can be controlled by the different dimensions in the 16D feature vector, and can also be controlled by the same dimension, which can be seen in the **Figure 12** for the ‘Width feature’. Secondly, after making 16D capsule’s features interpretable, we tried to find the relationship between voxels and these interpretable features. In order to reconstruct image stimuli, the high-level capsule’s features are firstly predicted through the three layers’ neural network using voxels. Therefore, we can obtain the numerical relationship between the output (predicted high-level capsule’s features) and input (voxels) based on the gradients of the three layers’ neural network using back propagation. So, we can obtain which voxels are more active than others for specific dimension in capsule feature vector, and further for interpretable features according to the values of the gradients. In this way, we presented the relationship between voxels and specific interpretable features. As shown in the **Figure 13**, we can see that different voxels in selected 100 voxels indeed contribute differently on specific interpretable features, and showed different sensitivity for different interpretable features. In addition, we can see that each interpretable features are decided by some voxels instead of one voxel that will bring more overfitting and less generalization considering inevitable noise during data acquisition. Based on the two steps, we can make voxels interpretable in specific image features, which proves the effectivity and generalization of the proposed method.

## Discussion

### CapsNet Architecture Performs Better in Extracting Visual Features

In a regular CNN, there are generally several pooling layers. Unfortunately, these subsampling layers tend to lose information for invariance, such as the precise location and pose of objects. It’s really not a big deal if you want to classify whole images, but it makes it challenging to perform accurate image segmentation, object detection, and other tasks which require precise location and pose information. Visual reconstruction is exactly the problem that needs to rely on complete characteristic information, which requires the equivariance instead of invariance when feature representation. The CapsNet architecture can be exactly fit for the problem, which benefits from the concept of capsule, dynamic routing, and reconstruction regularization loss. In addition, it is obvious that human can simultaneously accomplish many different tasks such as image recognition, image object location, and object pose detection after looking at one image only once. Different tasks always need different characteristics of images and it can conclude that visual information processing in human visual cortex also requires the equivariance. The equivariance ensures that the location, scale, pose, and some other detailed information be preserved instead of discarding. The similarity demonstrated that the new CapsNet architecture accords well with the human visual mechanism. Other architecture such as prevailing CNN does not have the equivariance, because they aimed at invariance, abstracting, and continues hierarchical abstracting in the process of forward propagation. Our results about encoding during selecting voxels and reconstruction results based on fMRI data both proved the significance of the new CapsNet architecture, which is very promising for visual reconstruction including more complex natural image reconstruction.

### The Importance of Selecting Voxels

As we know, selecting too many voxels will inevitably introduce more noise and not selecting enough voxels will miss some necessary information, which can both influence the quality of visual reconstruction. So, selecting voxels is an important procedure and challenging problem in visual reconstruction. On one hand, from the mean correlation coefficient of encoding based on top-k and R2 selection, we can find that the means of R2 nearly selected the most of top-k voxels. The second-stage training avoid the overfitting and the 10% higher on SSIM is partly attributed to the performance of selecting voxels, which indirectly indicate the importance of selecting voxels. On the other hand, we employed the much simple linear regression for the encoding model to select voxels, and the distance between the theoretical reconstruction and visual reconstruction still remain wide, which indicates that we need to select better voxels to optimize the mapping from fMRI data to high-level capsule’s features. How to select the more of top-k voxels and employing non-linear encoding may be next choices.

### Two-Stage Training Method

We realized the reconstruction with the two-stage training by introducing the new CapsNet architecture that provides the equivariance. We tried to add the third stage of fine tuning that optimize the overall network including the CapsNet and the mapping from fMRI data to capsule’s features, however, the results did not present the prospective effects. We think that the limited training samples (less than 100) are not suitable for the jointly training. However, the jointly training or end-to-end training is indeed a good direction from the development of computer vision, and the end-to-end training for visual reconstruction based on the CapsNet and more samples may attract more attention in the future.

### Generalization Analysis

Generalization is a matter of great concern in the fMRI based studies. In the section “Demonstration of Voxels Related to Specific Features”, voxels are interpreted in the specific features, which indirectly indicates the generalization of our proposed CNAVR method. However, because of the limit of subject, equipment, time, and so on, the generalization is hard to directly validate by performing exhaustive experiments. So, the generalization analysis for different subjects and category of image stimuli is additionally added to illustrate the application in other condition. On one hand, it is well known that most decoding models need training again for different subjects, and it is hard for one model whose parameters are kept fixed to obtain good reconstruction from different subjects’ fMRI, because of the significant individual difference in human brain. So, one architecture with different parameters for different subjects is acceptable. While dealing with a new subject, our proposed method is expected to obtain good reconstruction and needs to train again by the way of two-stage training in the section “Materials and Methods”. Certainly, the acquisition and pre-processing of fMRI data for different subjects usually need to keep the same. On the other hand, current stimuli used to perform visual reconstruction can be roughly divided into two categories (simple image stimuli and natural image stimuli). For simple image stimuli similar with the data used in this study, the mass of images and corresponding category labels are required to learn feature representation of equivariance through the CapsNet in the section “Training Image Feature Representation of Equivariance”. As shown in the study, we firstly train the network by using digit images from LeCun (1998). Luckily, the training does not need a large number of image stimuli with corresponding fMRI data of human visual cortex, which guarantees the generalization of the CNAVR method. Image stimuli are usually selected from public image dataset, and it is easy to collect a number of similar images with category labels. Therefore the CNAVR is expected to obtain good reconstruction for new simple image stimuli. In addition, the CapsNet is promising for reconstruction of natural image stimuli, a more challengeable problem, because reconstruction of more complex image stimuli requires equivariance more, and more complex image stimuli have more complex characteristics and patterns. However, the CNAVR method currently has difficult in reconstructing complex natural image stimuli, because the equivariance from natural images to capsule features is hard for the CapsNet to learn, which is a public problem in the CapsNet. The next key step is to solve the problem of how to better preserve the equivariance when feature representation. It is worth noting that we are first to introduce the CapsNet architecture into visual reconstruction, and it is no doubt that there is long way to improve the generalization further.

## Conclusion

This paper firstly introduced the new CapsNet architecture for visual reconstruction, inspired by the equivariance of information processing in human visual cortex. We proposed the CNAVR method that provides the equivariance when feature representation. Selecting voxels to reduce the dimensionality of fMRI data and learning the mapping from fMRI data to the capsule’s features are the two key stages in visual reconstruction. Based on the capsule’s features of equivariance, the performance of the two key stages are guaranteed. In comparison to the state of the art methods, the CNAVR exceeded by about 10% than the state of the art in the most important SSIM metric. These results demonstrated that our CNAVR better accords well with the human visual cortex. In addition, we analyzed the voxels in specific interpretable image features. To the best of our knowledge, this paper is the first to study visual image reconstruction via promising capsule network. Next, in order to achieve better visual reconstruction especially for complex images or videos, the exploration of CapsNet may spring up. There is no doubt that it’s still a start, but a promising start.

## Author Contributions

KQ proposed the idea of reconstruction based on the capsule network and writing. CZ contributed to the implementation of the idea. LW contributed to the idea of reconstruction based on the capsule network. JC designed the procedures of two-stage training. LZ contributed to the preparation of the article, figures, and charts. LT contributed to all stages of the research project and writing. BY introduced the perception of invariance and equivariance for visual reconstruction.

## Conflict of Interest Statement

The authors declare that the research was conducted in the absence of any commercial or financial relationships that could be construed as a potential conflict of interest.

## Footnotes

1 http://artcogsys.com/data/69dataset.mat
